# Characterization of microRNAs expression during maize seed development

**DOI:** 10.1186/1471-2164-13-360

**Published:** 2012-08-01

**Authors:** Mingming Kang, Qian Zhao, Dengyun Zhu, Jingjuan Yu

**Affiliations:** 1 State Key Laboratory of Agrobiotechnology, College of Biological Sciences, China Agricultural University, Beijing, 100193, China

## Abstract

**Background:**

MicroRNAs (miRNAs) are approximately 20-22 nt non-coding RNAs that play key roles in many biological processes in both animals and plants. Although a number of miRNAs were identified in maize, the function of miRNA in seed development was merely discussed.

**Results:**

In this study, two small RNA libraries were sequenced, and a total reads of 9,705,761 and 9,005,563 were generated from developing seeds and growing leaves, respectively. Further analysis identified 125 known miRNAs in seeds and 127 known miRNAs in leaves. 54 novel miRNAs were identified and they were not reported in other plants. Additionally, some miRNA*s of these novel miRNAs were detected. Potential targets of all novel miRNAs were predicted based on our strict criteria. In addition to deep-sequencing, miRNA microarray study confirmed the higher expression of several miRNAs in seeds. In summary, our results indicated the distinct expression of miRNAs during seed development.

**Conclusions:**

We had identified 125 and 127 known miRNAs from seeds and leaves in maize, and a total of 54 novel miRNAs were discovered. The different miRNA expression profile in developing seeds were revealed by both sequencing and microarray studies.

## Background

MiRNAs are known as regulators that control various types of biological functions in eukaryotic cells. Most miRNA genes are transcribed to primary miRNAs (pri-miRNAs) by RNA polymerase (RNApol) II, while other miRNAs may be transcribed by RNApol III [1]. Then the pri-miRNAs are processed to single-stranded stem-loop precursors (pre-miRNAs) by Dicer protein in animals or Dicer-like (DCL) in plants [2-4], and pre-miRNAs are further processed to miRNA:miRNA* duplexes. The miRNA:miRNA* duplexes are methylated by HEN1 on the 3’ terminal [5] and exported to cytoplasm by export factors like Exportin-5 in animal or HASTY in plant [6,7], then the mature miRNAs and miRNA* are produced. Mature miRNAs are loaded into RNA-induced silencing complex (miRISC) that contains ARGONAUTE (AGO) protein to guide the cleavage of target mRNAs that are complementary to the miRNAs, while the miRNA*s are degraded gradually [1,8,9]. Plant miRNAs were first reported in 2002 in *Arabidopsis*[2,10]. They are usually 20-22 nt in length and their precursors have various length ranging from 60 nt to longer than 350 nt. Unlike miRNAs in animals, plant miRNAs have near-perfectly complementary sequences to their targets sites [1,11]. There are two major regulation mechanisms of plant miRNAs, known as mRNA cleavage and translational inhibition. The positions of 10 and 11 of miRNAs are critical for the mRNA targeting and cleavage at 3’ UTR region [12]. Beside mRNA cleavage, plant miRNAs can also function through translational inhibition that can decrease the level of target proteins [13,14]. To date, most identified targets of plant miRNAs are transcription factors that are involved in many biological processes such as developmental patterning, cell differentiation and stress response [1,15-17]. Recently, the roles of plant miRNAs were widely studied in various organisms including *Arabidopsis*, rice, maize and sorghum.

MiRNAs are involved in many regulatory pathways that controls seed development, and miRNA loss-of-function may lead to developmental defects or even lethality [18]. In *Arabidopsis*, miR156 targets *SPL10* (*Squamosa Promoter-Binding Protein-Like 10*) and *SPL11*, and over accumulation of these targets leads to abnormal cell divisions [18]. Plants expressing miR160-resistant *ARF17* (*Auxin Response Factor 17*) may cause abnormal embryo symmetry [19]. Overexpression of miR164 reduces the *CUC1*/*CUC2* (*CUP-SHAPED COTYLEDON*) transcripts and results in cotyledon development defect [20]. The sequences of miR159 and miR319 are nearly identical and both can affect seed size. MiR172 targets several *APETALA2* (*AP2*)*-like* transcription factors that control seed mass and yield [21]. Therefore, further focus on developmental roles of miRNAs may reveal more detailed functions of miRNAs in seed development.

Currently, there are 319 discovered maize miRNAs in the miRNA database miRBase (Release 17, April 2011) [22], most of which is primarily identified by similarity comparison to mature miRNAs from other plant species [23,24]. The genome-wide analysis of miRNAs and their targets in maize had proved the importance of miRNAs in gene regulation network throughout plant development [24]. To further study these miRNAs in seed development, we sequenced two sRNA libraries from developing seeds and young leaves, leading to the identification of 125 and 127 known miRNAs in seeds and leaves, respectively, and the discovery of 54 novel maize miRNAs. Small RNA deep-sequencing is a ideal way for miRNA profiling due to the high throughput comparing with other approaches. We further characterized the miRNA expression by miRNA microarray study, and both sequencing and microarray data uncovered the similar expression pattern of several known miRNAs in developing seeds.

## Results and discussion

### Small RNA sequencing

**Figure 1:**
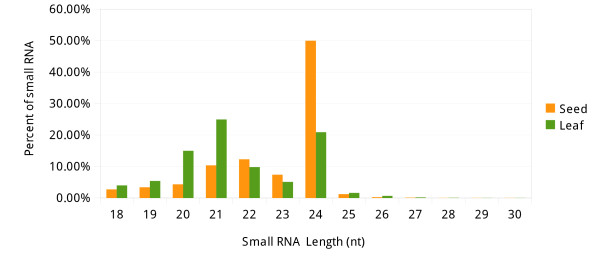
Total reads of 18-30 nt small RNAs.

To study the role of miRNA during seed development, a small RNA library from five stages of immature seed (see Methods) was generated and sequenced by Illumina’s Genome Analyzer. After removal of low quality reads and adaptor sequences, a total of 9,705,761 reads representing 5,396,301 unique reads from 18 to 30 nt in length were obtained. The most sequenced sRNAs were 24-nt in length (50%), which was the feature that some siRNAs had, followed by 22-nt (12.3%) and 21-nt (10.4%), which were the length of canonical miRNAs (Figure 1). The abundance of unique reads was remarkably different, however. For example, zma-miR168a/b, with the total count of 131,141, is the most sequenced read, but around 82% (4,429,004) of total signatures was sequenced only once, indicating that the small RNA population in maize might be highly complicated. Then, the reads were mapped to the maize genome (B73 RefGen_v2, release 5b.60) using Bowtie [25] with a tolerance of one mismatch. The results indicated that 271,104 reads matched perfectly to the maize genome, representing 87,873 unique sRNAs, and 162,106 reads had one nucleotide differed from the genome, representing 79,930 unique sRNAs. Approximately 3.76% unique reads matched other non-coding RNAs including rRNA (0.90%), tRNA (0.11%), siRNA (2.71%), snRNA (0.03%) and snoRNA (0.01%), which made up 12.12% of total sequenced reads (Table 1).

**Table 1:** Summary of small RNA sequencing

	**Seed**		**Leaf**	
	**Unique reads**	**Total reads**	**Unique reads**	**Total reads**
Non-coding RNAs				
rRNA	48,744 (0.90%)	496,691 (5.12%)	98,035 (4.70%)	1,923,961 (21.36%)
tRNA	6,190 (0.11%)	197,396 (2.03%)	13,441 (0.64%)	639,236 (7.10%)
siRNA	145,973 (2.71%)	476,425 (4.91%)	42,910 (2.06%)	173,554 (1.93%)
snRNA	1,828 (0.03%)	4,821 (0.05%)	1,169 (0.06%)	2,399 (0.03%)
snoRNA	557 (0.01%)	949 (0.01%)	504 (0.02%)	1,186 (0.01%)
Protein-coding RNAs				
exon	590,744 (10.95%)	1,201,276 (12.38%)	307,933 (14.74%)	810,348 (8.90%)
intron	747,218 (13.85%)	1,494,227 (15.40%)	310,191 (14.86%)	656,538 (7.29%)
Known miRNAs				
mature	226 (0%)	462,609 (4.77%)	250 (0%)	3,098,983 (34.41%)
mature star	97 (0%)	1,697 (0.02%)	131 (0%)	8,915 (0.10%)
Other sRNAs	3,854,724 (71.43%)	5,369,670 (55.32%)	1,313,335 (62.90%)	1,690,443 (18.77%)
Total	5,396,301 (100%)	9,705,761 (100%)	2,087,899 (100%)	9,005,563 (100%)

To see if the expression of miRNAs was different from other tissues, another small RNA library from young leaves was also sequenced and data were processed by the same procedure as described above. Unlike in seeds, the dominant reads in leaves were 21-nt sRNAs (25%), and 24-nt sRNAs only accounted for 20.9% compared to seeds. Other abundant signatures included 20-nt (15%) and 22-nt (9.8%) sRNAs (Figure 1). A total of 402,882 sRNAs matched perfectly to the genome and 59,559 sRNAs differed from genome by one nucleotide. Around 30.43% of total sRNAs were annotated as other non-coding RNAs (Table 1).

The sRNAs length distribution of the two libraries suggested a distinct sRNA population in seeds. As mentioned above, 24-nt sRNAs were highly accumulated in seeds (50%), which was consistent with two recent studies in maize [26,27].

### Identification and characterization of conserved miRNAs

**Figure 2:**
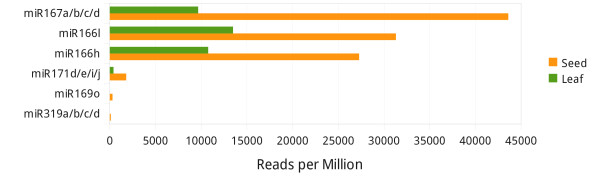
**More abundant conserved miRNAs in seeds.** MiR319a/b/c/d and miR169o were detected only in seeds.

Since miRNA plays a important role in plant development, there is a growing number of both mature and precursor miRNAs registered in the miRNA database miRBase (http://www.mirbase.org). There are currently 319 maize mature miRNAs and 170 miRNA precursors in the database (release 17, April 2011). To identify conserved miRNAs, small RNA sequences were aligned to maize mature miRNAs and precursors with perfect matches, and 125 conserved miRNAs belonged to 24 miRNA families and 127 miRNAs belonged to 25 miRNA families were identified in seeds and leaves, respectively. Despite the similar family number, these conserved miRNAs were generally more abundant in leaves (462,609 and 3,098,983 reads in seeds and leaves, respectively), indicating that the regulation network that involves miRNAs is more complicated in the vegetatively growing seedlings. However, there are miRNAs that were less expressed in young leaves but relatively rich in seeds. Our result indicated that the zma-miR319a/b/c/d and zma-miR169o were detected only in seeds, with 42 and 124 reads respectively, similar with miR319 expression in the developing seeds reported previously [26]. MiR319 targets several TCP transcription factors in *Arabidopsis*[28] and can affects seed size. Several studies also revealed that miR319c, rather than miR319a/b, played a important role in *Arabidopsis* leaf development [28-30]. In maize, miR319 was predicted to target several transcription factors including MYB and TCP domain proteins [24]. Most members of zma-miR171, zma-miR167 and zma-miR166 families not only had higher reads but the reads were higher in seeds (Figure 2). MiR167 targets several ARF transcription factors that are important in controlling seed dispersal. A recent study reported that miR166 was sequestered by AGO10 to prevent its loading to AGO1, consequently several *HD-ZIP III* transcription factors were suppressed and this was critical for shoot apical meristem development [31].

Zma-miR168 and zma-miR528 were the top sequenced in seeds and leaves, respectively (Additional file 1). Intriguingly, one target of miR168 was *AGO1* which was essential for miRNA maturation [15,32], and the interaction between miR168 and *AGO1* maintained proper embryo development. Besides, miR168 was also responsive for several salt-stresses in maize [33,34]. Recent studies suggested that miR528 showed significant repression under low nitrate condition in maize roots and shoots [34], while in *T. dicoccoides*, miR528 was down-regulated in leaves during drought stress [35]. Other miRNAs in high abundance include zma-miR164, zma-miR156 and zma-miR827, which were more than 1,000 reads in both two tissues. In summary, these suggested a developmental and tissue-specific expression of miRNAs, and therefore the more abundant miRNAs in seeds should have key functions in regulating seed development.

### Diverse expression pattern of small RNAs in seeds and leaves

As mentioned above, seeds and leaves had preferential expression of small RNAs, these might be mainly due to the different developmental stage they stayed in. Previous studies suggested that in the early stage of embryogenesis, miRNAs inhibited genes from prematuration to preserve proper developmental stage [18]. In the immature seeds, a variety of environment and stress-response genes were relatively less expressed, and a number of these genes were the targets of miRNAs, therefore the regulation network that involved miRNAs could be less complicated than in other developmental stage. Whereas the ever-growing seedings need more nutrition, and had to confront all substantial stresses and adjust themselves to survive. Therefore, regulatory sRNAs in leaves should be more complicated. Previous studies also suggested that, the miRNA held at a relatively lower level in plant seeds rather than in other tissues [26,36]. The similar situation was also found in animal, as the targets of several miRNAs were highly expressed in embryo than in other tissues, and the miRNAs in turn were in low level in embryo [37]. The sequencing data in this study showed a similarity with those discussed above. Total reads of conserved mature miRNAs were more than 7-fold higher in leaves (7,700,465 RPM) compared with those in seeds (1,067,863 RPM), which was consistent with previous studies [26,36]. In the seed sequencing library, around 50% of signatures were 24-nt in length (Figure 1), and 20-22 nt sRNAs only took up 16.8% of the population. In contrast, the most signatures in leaves were 21-nt sRNAs that accounted for 25% of total reads. Previous study indicated that a number of seed sRNAs were most likely derived from repeat-associated siRNAs (rasiRNAs) [36]. In our case, the same situation was also found by scanning the folded clusters of sRNAs by RepeatMasker. Another siRNA, called trans-acting siRNA (tasiRNA), was involved in controlling seed size, and both this tasiRNA and its target were conserved in rice and maize [38]. A recent study suggested that the siRNA-mediated DNA methylation was enhanced in seeds [39]. These findings indicated the siRNA’s important role in seed development. In summary, our study showed that miRNAs were lower expressed in seeds considering both total and unique number, indicating that miRNA expression was still in initialization in seeds.

### The role of miRNA*s in plant development

During miRNA biogenesis in plants, miRNA:miRNA* was spliced by DCL1 to produce functional mature miRNA, while miRNA* was assumed to be gradually degraded [1]. The established model of selection of functional mature strand was based on the thermodynamic structures of miRNA:miRNA* duplexes [40], whereas the unstable strand was so-called “miRNA*” and assumed to degrade to a nearly undetected level [41]. Nevertheless, previous research suggested that miRNA*s could also accumulate to a considerable level and down-regulate their target genes in both plants and animals [42-45]. In mouse, both the mir-30e-5p and mir-30e-3p could suppress the expression of their targets [46]. More recently, new evidence of miRNA*-mediated mRNA cleavage was found in *Medicago truncatula* by degradome sequencing [47]. The same situation was found in maize as well [48], indicating the yet less discovered function of miRNA*. Besides, due to the over-expression of miRNA precursors, the enrichment of miRNAs or miRNA*s was proposed to affect their precursors via a feedback pathway. We noticed that in our sequencing result, a few of miRNA*s had been highly sequenced in both two sequencing libraries (Additional file 1). The total reads of miR408b* and miR396a*/b* were greater than the corresponding mature sequences, and both showed higher abundance in young leaves. MiR408b* had the max reads of 5,716 in leaves, which was even higher than the mature zma-miR408b (4,699 reads). Similarly, miRNA396a*/b* were also more sequenced (223 reads) than the mature miRNA (99 reads) in seeds. Since miRNA* may have the same function as mature miRNA [49], it is possible that these miRNA*s can be *de facto* miRNAs as well. In summary, these miRNA genes should have alternative expression preferences according to the special developmental stage and environment.

### Validation of known miRNA expression by miRNA microarray

**Figure 3:**
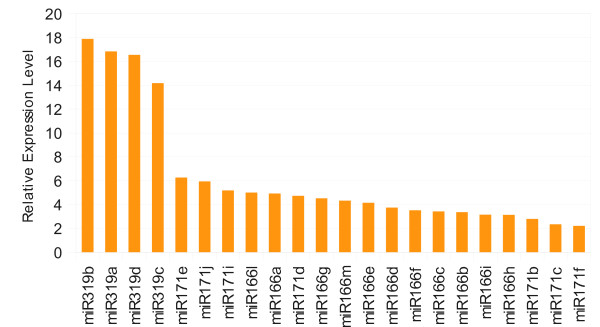
Highly expressed miRNAs in seeds through miRNA microarray.

To further study the expression of conserved miRNAs in maize seeds, we carried out miRNA microarray analysis. The result indicated that, compared to leaves, all 4 miRNA members from miR319 family had a average of 16 times detection signals higher in seeds, which further confirmed our sequencing result (Figure 3). Moreover, miRNAs of at least 2 times expression level higher in seeds were all from miR319, miR171 and miR166 family (Figure 3, Additional file 2), indicating the important roles of these miRNAs in seed development. MiR166 was predicted to target basic-leucine Zipper (bZIP) genes in maize [24], which could regulated many processes such as seed maturation, stress signalling and flower timing [50]. miR171 was predicted to target GRAS transcription factor that controlled gibberellic acid (GA) signaling and phytochrome A signal transduction [51]. The more abundant miRNAs in leaves were miR399a/c/d/e, miR408, miR159c/d, miR156k/j, miR160a/b/c/d/e and miR164a/b/c/d, while the expression of miR399e was nearly 10-fold higher than that in seeds (Additional file 2). MiR156 targeted SBP transcription factor [52,53], which affected shoot maturation in *Arabidopsis*, miR159c/d and miR164 were predicted to target MYB domain transcription factors [24,54], and miR160 targeted ARF transcription factors [55,56]. Taken together, the highly expressed miRNAs and the target types of these miRNAs indicated the developing plants had a more sophisticated gene regulation mechanism and thus needed a number of regulators such as miRNAs.

### Identification and characterization of novel maize miRNAs

**Figure 4:**
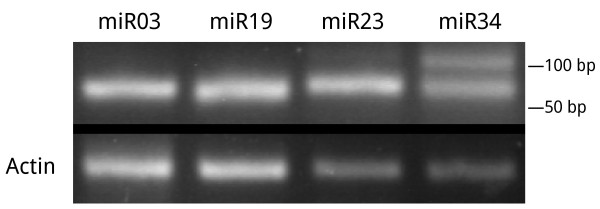
**Novel miRNAs validated by stem-loop RT-PCR.** Four novel miRNAs (miR03, miR23 and miR34 from seed, miR19 from leaf) were validated by RT-PCR and sequencing. 30 cycles of standard PCR was used to amplify Actin. A non-specific band was found in the miR34 PCR product (upper).

In plants, the pri-miRNA transcript was spliced twice by DCL RNase [1], and a hairpin-like miRNA precursor was produced after the first cleavage. Since the special structure, secondary structures of clusters were predicted by a RNA folding program RNAfold [57], sequences that matched to the genome from seeds and leaves totally folded 61,399 and 18,106 clusters, respectively. Previous studies suggested that the miRNA precursors had a average minimal folding free energies index (MFEI) of 0.97, which was significantly higher than other non-coding RNAs such as mRNAs (0.62-0.66), rRNAs (0.59) and tRNAs (0.64), and 90% of miRNA precursors had an MFEI greater than 0.85 while no other RNAs had this feature [58]. To validate these clusters, the MFEI of these clusters was checked that it should be no less than 0.85, finally 25 and 33 clusters were regarded as novel miRNA precursors in seeds and leaves respectively (Additional file 3), while the most abundant miRNAs were regarded as novel miRNAs. These novel miRNAs can be further grouped into 43 miRNA families from both seeds and leaves, representing 54 individual novel miRNAs (Table 2, Additional file 4).Four novel miRNAs were experimentally validated by stem-loop RT-PCR and sequencing (Figure 4).

**Table 2:** Summary of newly identified miRNAs

**miRNA**	**Sequence**	**Length**	**Abundance**		**Pre-miRNA**	**Conservation**
	(5’-3’)	**(nt)**	**Seed**	**Leaf**	**Position**^**1**^	
zma-miR01	AAAAAGCCAGAACGATTTATGA	22	15	-	Intron	
zma-miR02a	AAGCAAGGATAATGGAGGGGA	21	9	-	Intron	
zma-miR02b	AAGCAAGGATAATGGAGGGGA	21	9	-	Intron	
zma-miR03	ACCGATCGGGAGAACCGGAGA	21	-	37	Overlap	
zma-miR04	ACGGTGTTGTGTCAGGGGGGT	21	6	-	Intergenic	
zma-miR05	AGAACCGGAGAGCTAGAGGG	20	-	5	Overlap	
zma-miR06	AGAGGAGATTGAAGGGGCTAG	21	6	-	Intergenic	
zma-miR07	AGAGGATCTATGGTGGAGGAA	21	5	-	Intron	
zma-miR08	AGATATGGTAGAGGGGCCTAA	21	7	-	Intergenic	*O. sativa*
zma-miR09	AGCTATGAACGTCTGGATGCA	21	-	5	Intergenic	
zma-miR10	AGTGTTTGGTTAGATGGAATAG	22	-	26	Intergenic	
zma-miR11	ATACTAGGAGTGAAGGGATCA	21	8	-	Intron	
zma-miR12	ATATATGTGGGTTGGGATTAAT	22	5	-	Intron	
zma-miR13	ATCACAGGAGGATTGGAGGAG	21	9	-	Intron	
zma-miR14a	ATGGAGGGGATTGAGGGGCTA	21	-	9	Intergenic	
zma-miR14b	ATGGAGGGGATTGAGGGGCTA	21	-	9	Intergenic	
zma-miR14c	ATGGAGGGGATTGAGGGGCTA	21	-	9	Intergenic	
zma-miR15	ATGGTGCATTGACTTGGTCAA	21	-	5	Intron	
zma-miR16	ATTGTAGTGGATTGAGAGGGA	21	8	-	Intergenic	
zma-miR17	ATTTTTGAAGGAAGGAAAGC	20	9	-	Overlap	*S. bicolor*
zma-miR18a	CAAAGAGAATTGAGGGGGCTA	21	10	7	Intergenic	
zma-miR18b	CAAAGAGAATTGAGGGGGCTA	21	-	7	Intergenic	
zma-miR18c	CAAAGAGAATTGAGGGGGCTA	21	-	7	Intergenic	
zma-miR18d	CAAAGAGAATTGAGGGGGCTA	21	10	7	Intergenic	
zma-miR19	CCAACAGGATATTGGGTATTTC	22	169	-	Intergenic	*O. sativa*
zma-miR20	CGCAGCGTTGATGAGCCAGCCG	22	57	7	Intergenic	*S. bicolor*
zma-miR21	CGGCTCACCAGCGCTGCACTC	21	6	-	Intergenic	
zma-miR22a	CTGAAAAGTGTGGCGCGGTGT	21	-	9	Intergenic	
zma-miR22b	CTGAAAAGTGTGGCGCGGTGT	21	-	9	Intergenic	
zma-miR23a	GAGACAGACAACATATGTAGAA	22	-	26	Intron	
zma-miR23b	GAGACAGACAACATATGTAGAA	22	5	-	Intron	
zma-miR24	GAGCGCAGCGTTGATGAGCCAG	22	5	-	Intergenic	*S. bicolor*
zma-miR25	GGAGGAGATGGGAGTGGCTAA	21	12	-	Intergenic	*S. bicolor*
zma-miR26a	GTCACAGAAGTTGGGATGCAA	21	5	-	Intron	*S. bicolor*
zma-miR26b	GTCACAGAAGTTGGGATGTAA	21	-	13	Intron	*S. bicolor*
zma-miR27a	GTGATCACGGGAGATTGGAGA	21	-	7	Intergenic	
zma-miR27b	GTGATCACGGGAGATTGGAGA	21	48	-	Intergenic	
zma-miR28	TAGAGAGGATTAAAGTGGCTA	21	-	5	Intergenic	
zma-miR29	TAGCTCTTCCTGTTTGGATAT	21	5	-	Intergenic	*S. bicolor*
zma-miR30	TAGGGATCTATGGAGAGGAA	20	5	-	Intergenic	
zma-miR31	TCAACACACGTGGATTGCGGT	21	-	6	Intron	
zma-miR32	TCACAAGGGGATTGAAGAGGA	21	5	-	Intron	
zma-miR33	TCACTTTGGGATCACAGATAA	21	10	-	Intron	
zma-miR34	TCAGAAAATATGAACTTGAGA	21	-	19	Intron	
zma-miR35	TCATAAGGGGATAAACAACGC	21	-	5	Intron	
zma-miR36	TCGGGGTTAGAGGGGATTGAG	21	6	-	Intergenic	*O. sativa*
zma-miR37	TGAAAAGCTAGAACGATTTAC	21	5	-	Intron	
zma-miR38a	TGAAGAGAATTGAGGGGGCTA	21	17	-	Intergenic	
zma-miR38b	TGAAGAGAATTGAGGGGGCTA	21	17	-	Intron	
zma-miR39	TGGACAGGGAAATGAAGGGGA	21	16	-	Intergenic	
zma-miR40	TGGAGGGGATTGAGGGGCATA	21	-	8	Intergenic	
zma-miR41	TTAGATGGGATACATGAGAGG	21	-	5	Intergenic	
zma-miR42	TTAGTAGTTTTAGTTCTTTGC	21	5	-	Intergenic	*A. thaliana*
zma-miR43	TTTAGTGATCAGCTGGAGGTT	21	-	5	Intron	*S. bicolor*

^1^“Intron/Intergenic” indicates full-length of pre-miRNA localizes to that region, and “Overlap” indicates pre-miRNA overlaps with intron/exon or exon/intron (see Additional file 5).

**Figure 5:**
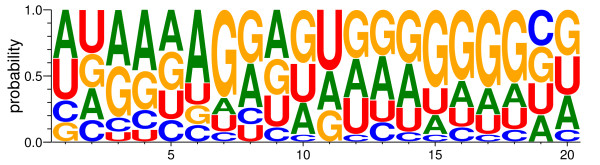
Nucleotide frequency of novel miRNAs.

As a further evidence, we had also detected some corresponding miRNA*s of novel miRNAs, though these miRNA*s were low in abundance (Additional file 4). The nucleotide composition analysis showed that more than 20% of novel miRNAs start with U, which is statistically the typical property of mature miRNAs [59,60], and the overall nucleotide composition showed higher percentage of A and G (Figure 5). The genomic position analysis of these novel miRNA precursors showed that most of pre-miRNAs (31 out of 54 pre-miRNAs) localized to the intergenic regions, more than one third of pre-miRNAs (20 out of 54 pre-miRNAs) were located within the intronic regions of protein-coding genes, and 3 pre-miRNAs overlapped with adjacent intron/exon (2) or exon/intron (1) (Table 2, Additional file 5). The exact location of novel miRNA genes may differ a little since the pri-miRNAs were not cloned experimentally. To see if the newly identified miRNAs were conserved in other plants, miRNA sequences were blastn searched against the genome sequence of *Arabidopsis*, rice and sorghum (see Methods). The result indicates that 8 miRNAs were conserved in sorghum, 3 miRNAs conserved in rice and 1 miRNA conserved in *Arabidopsis* (Table 2).

### Target prediction of novel miRNAs

Since the miRNA and target duplexes are near-perfectly matched in plants, it is possible to find targets by computational approach. Among the 54 newly identified miRNAs, 41 had predicted targets that fulfilled the criteria described previously [61]. To make the prediction more reliable, we didn’t allow any mismatches at miRNA position 2-12 with a max of 3 discontinuous mismatches in the pairing region (see Methods). The additional screening suggested that 28 miRNAs had the almost perfectly paired targets (55 in total), while 53 out of the 55 targets had functional annotations in the InterPro entries [62] (Additional file 6). The predicted targets belonged to various classes of molecular functions, such as DNA/RNA binding protein, protein kinase and other enzymes, or of biological processes, such as thiolase, cation transporter and lipid metabolic enzymes, or of several cellular components, indicating the miRNA’s extensive role in gene regulation network. As shown in additional data, several miRNAs targeted transcription factors, consistent with their functions reported earlier. Zma-miR16 and zma-miR37 were predicted to target bZIP-1 transcription factor, zma-miR24 targeted both MADS-box and K-box transcription factor, and zma-miR19a and zma-miR20a targeted genes with MYB DNA-binding domain (Table 3, Additional file 6). All these genes had important regulation roles throughout plant development [54,63,64].

**Table 3:** Predicted novel miRNA targets with DNA-bind domain

**miRNA**	**Target ID**	**InterPro ID**	**InterPro description**
zma-miR04	GRMZM2G171365	IPR002100	Transcription factor, MADS-box
		IPR002487	Transcription factor, K-box
zma-miR14a/b/c	GRMZM2G438293	IPR004827	Basic-leucine zipper domain
		IPR011616	bZIP transcription factor, bZIP-1
zma-miR18a/b/c/d	GRMZM2G315506	IPR001005	SANT/Myb domain
		IPR006447	Myb DNA-binding domain, plants
zma-miR37	GRMZM2G118870	IPR004827	Basic-leucine zipper domain
		IPR001841	Zinc finger, RING-type
		IPR013083	Zinc finger, RING/FYVE/PHD-type
zma-miR40	GRMZM2G155980	IPR004330	FAR1 DNA binding domain
		IPR006564	Zinc finger, PMZ-type
		IPR007527	Zinc finger, SWIM-type

## Conclusions

We had studied the miRNA expression profile during maize seed development by combining small RNA sequencing and miRNA microarray. By sRNA sequencing, 125 and 127 conserved miRNAs were identified in the developing seeds and young leaves, respectively. Furthermore, 54 novel miRNAs were identified which were not reported before, and potential targets were also predicted with strict criteria as described. Both deep-sequencing and miRNA microarray suggested that miR319, miR166 and miR167 were highly expressed in the developing seeds. In addition, we found miRNA408b* and miR396a*/b* had been accumulated to a level higher than their mature sequences.

## Methods

### Small RNA libraries construction and RNA sequencing

Maize (*Zea mays*) inbred line B73 was used in this study. Plants were grown under natural conditions. Immature seeds at 10, 15, 20, 25 and 30 days after pollenation (DAP) were collected seperately, the young leaves from seedlings grown in soil at 22°C with 16 h/8 h light cycle were obtained 3 weeks after germination. All tissues mentioned above were frozen in liquid nitrogen immediately after collection and stored at -80°C for further use. Total RNAs from immature seeds at 10, 15, 20, 25 and 30 DAP, and young leaves were extracted separately using TRIzol reagent (Invitrogen) and the integrity was checked by 1% agarose gel. The seed RNAs were then mixed with the same amount. Small RNA library construction was carried out as described [65]. Briefly, 16-30 nt small RNAs were gel-purified from 15% PAGE gel, 5’ and 3’ adaptors were added and followed by RT-PCR using adaptor-specific primers. The PCR products were isolated, gel-purified and used for cluster generation. Sequencing was performed using Illumina’s Genome Analyzer (Illumina Inc., USA). Clean reads were generated after filtering adaptor sequences and removal of low quality reads.

### Identification of conserved and novel miRNAs

Maize genome sequences with TE repeats masked and cDNA sequences (B73 RefGen_v2, release 5b.60) were downloaded from maizesequence.org (http://www.maizesequence.org). Mature miRNAs and precursors were retrieved from miRBase.org (Release 17, http://www.mirbase.org) [22], other non-coding RNAs (including rRNAs, tRNAs, snoRNAs, etc) were obtained from Rfam database (Rfam 10.1, http://rfam.sanger.ac.uk). Conserved miRNAs were identified by aligning to the registered maize miRNAs in miRBase database (Release 17, http://www.mirbase.org), sequences with perfect matches were regarded as conserved miRNAs. To predict novel miRNAs, a previously reported workflow was carried out as the initial step [66,67]. Known mature, mature star and harpin sequences, as well as transcriptome libraries including maize cDNA and Rfam, were filtered out from clean reads after alignment. The remaining reads were mapped to maize genome using Bowtie [25] with no more than one mismatch. Sequences that didn’t match the genome were discarded. The filtered reads were then clustered and secondary structures of miRNA clusters were checked by RNAfold (http://www.tbi.univie.ac.at/RNA/). RepeatMasker (http://www.repeatmasker.org) was used to remove repetitive sequences from clustered loci. Next, the MFEI values of the remaining clusters were checked. As previously studied, the MFEI of most miRNA precursors was greater than 0.85, which was notably higher than other non-coding sRNAs. MFEI can be calculated as MFE/(precursor length)×100/(G+C)%. Only clusters with the MFEI value greater than 0.85 were considered [58]. Then, the location of reads was checked to filter the ones that mapped on the loop region of corresponding cluster. Finally, the most abundant reads that were 20-22 nt in length with no less than 5 reads were regarded as novel miRNAs. To further validate the candidate miRNAs, we searched for the miRNA*s by blastn with 2-nt 3’ overhangs to the mature miRNAs. The genome locations of novel miRNA precursors were annotated by comparing the precursor position with the latest maize genome annotation (5b.60, www.maizesequence.org). The sequencing data were deposited at NCBI Gene Expression Ominibus (GEO, http://www.ncbi.nlm.nih.gov/geo/) under accession number GSE37551.

To study the cross-species conservation of novel miRNAs, genome sequences of *Arabidopsis*, rice and sorghum were obtained from http://www.arabidopsis.org, http://www.jcvi.org and http://www.phytozome.net. Sequences of no more than 4 mismatches with miRNA were reserved by blastn search against the genome sequences. Considering the plus/minus strand of pre-miRNA and 5p/3p location of mature miRNA, genome sequences of -50 to +250 and -250 to +50 (for plus strand), or -250 to +50 and -50 to +250 (for minus strand) were extracted and inverted repeats (IRs) were retrieved by EMBOSS einverted [68]. Then these IRs were filtered by the criteria for pre-miRNAs to obtain conserved miRNAs.

### Validation of novel miRNAs by stem-loop RT-PCR and sequencing

A previously reported stem-loop RT-PCR method was adopted for novel miRNA detection [69,70]. Total RNA from seed or leaf was extracted as described above. 800 ng RNA template was reverse-transcribed to cDNA by M-MLV reverse transcriptase (Promaga) using specific stem-loop RT primer. PCR was performed with the following procedures: 94°C for 5 minutes; 30 cycles of 94°C for 30 seconds and 60°C for 1 minutes. The PCR products were analysed by 4% agarose gel and purified by TIANgel Mini Purification Kit (Tiangen, Beijing), then ligated to pMD19-T vector (TaKaRa, Dalian China) and transformed to *E.coli* DH10B. Sequencing was carried out by Invitrogen (Shanghai). All primers used can be found in Additional file 7.

### Novel miRNA targets prediction

To predict potential targets of newly identified miRNAs, maize Filtered Gene set (release 5b.60) and annotation data were downloaded from http://maizesequence.org. Currently there were nearly 40,000 entries in the filtered set. We adopted a modified scoring method for target prediction as described [61,71]. Basically, targets should fulfill the following criteria: no more than 3 mismatches between miRNA and target, no mismatches at position 10 or 11 of miRNA, no more than 2 consecutive mismatches in position 2-12 of miRNA, and the MFE ratio of miRNA:target duplexes and miRNA:target-binding site duplexes should be greater than 0.75 [61,72]. More strictly, we further selected a subset from these potential targets with no mismatch allowed in position 2-12 and no adjacent mismatches throughout the miRNA:target binding site. The functional annotations of predicted targets were then retrieved by using InterPro as described [62].

### MicroRNA microarray hybridization

Mature miRNA sequences in miRBase (Release 12, http://www.mirbase.org) were downloaded for probe design. All probes were complementary to the mature miRNA sequences. A total of 7,815 probes (including 96, 203 and 275 mature miRNAs from maize, *Arabidopsis* and rice respectively, and other miRNAs from various organisms) were synthesized. Probes were then poly(T)-concatenated to 40 nt and 5’-amino-modifier C6 modified to strengthen their stability on aldehyde-modified chip surface. After dissolved in 40 *μ*M spotting solution, all probes were printed triply onto the activated chip sulface. GeneChip^*®*^ microRNA microarray was used for mature miRNA expression analysis (Affymetrix, USA). Total RNAs were isolated using mirVana^*®*^ RNA Isolation Kit (Ambion, USA), 1 *μ*g of total RNA containing low molecular weight (LMW) RNAs were poly(A)-tailed and biotin-labeled by FlashTag^TM^ Biotin RNA Labeling Kit for Affymetrix GeneChip^*®*^ miRNA Arrays. Hybridization, Wash and Stain Kit was used for hybridization, washing and staining according to the supplier’s instructions. Hybridizations were scanned by GeneChip^*®*^ Scanner 3000 (Affymetrix) and signals were normalized using Affymetrix Microarray Suit (version 5.0). The original microarray data were submitted to NCBI GEO under accession number GSE37322.

## Competing interests

The authors declare that they have no competing interests.

## Author’s contributions

JY conceived and designed the research. MK prepared the samples, performed the analysis and wrote the original manuscript. QZ managed the experimental condition. DZ managed the plant materials. JY revised thoroughly the manuscript and finalize the manuscript. All authors read and approved the final manuscript.

## Supplementary Material

Additional file 1 **Conserved maize miRNAs expression level.** The total reads of conserved miRNAs and corresponding miRNA*s, sorted by miRNA names. Expression difference were shown in column 4. Click here for file

Additional file 2 **Expression of conserved miRNAs validated by miRNA microarray.** The detailed normalized signals in the microarray experiment, including whole probes. Click here for file

Additional file 3 **Secondary structure of novel miRNA precursors.** Predicted secondary structures of newly identified miRNAs, mature miRNA sequences were shown in red, while the miRNA* sequences were shown in blue, if any. Click here for file

Additional file 4 **Novel maize miRNAs identified in this study.** Detailed information of newly identified miRNAs. Click here for file

Additional file 5 **Genome locations of novel miRNA precursors.** The genome locations of novel miRNA precursors based on the current annotation of maize genome (5b.60). Click here for file

Additional file 6 **Potential targets of novel miRNAs.** The filtered set of targets that had no mismatch in position 2-12 and no consecutive mismatch, filtered from the original set that passed the criteria described above. Annotations were retrieved from the InterPro database. Click here for file

Additional file 7 Primers used in this study. Click here for file
