# Supplementary material for: Characterization of microRNAs expression during maize seed development
Source: BMC Genomics. 2012 Aug 1;13:360. doi: 10.1186/1471-2164-13-360 (PMC3468377; doi:10.1186/1471-2164-13-360)

Additional Figure 1: Secondary structure of novel miRNA precursors

Mature miRNAs are highlighted in red color, and miRNA*s in blue, if any.
Zma-miRxx(*) indicates the detection of corresponding miRNA*.

zma-miR01

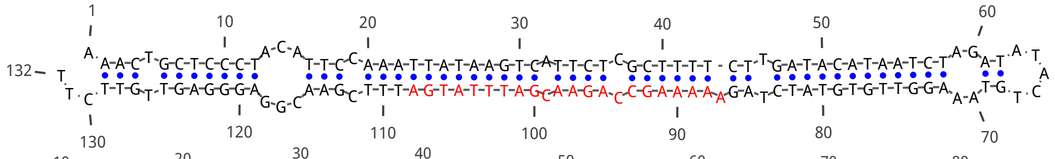

zma-miR02

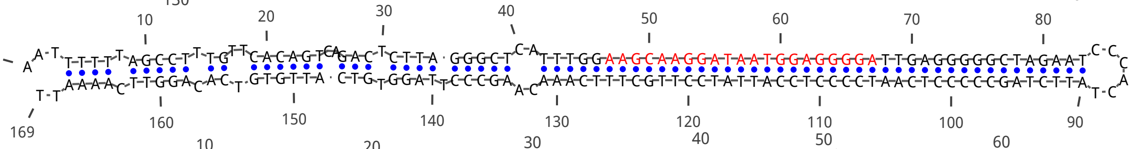

zma-miR03(*)

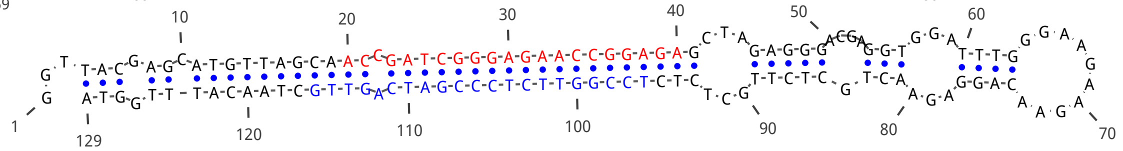

zma-miR04(*)

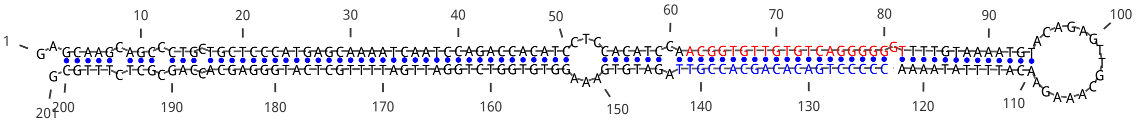

zma-miR05

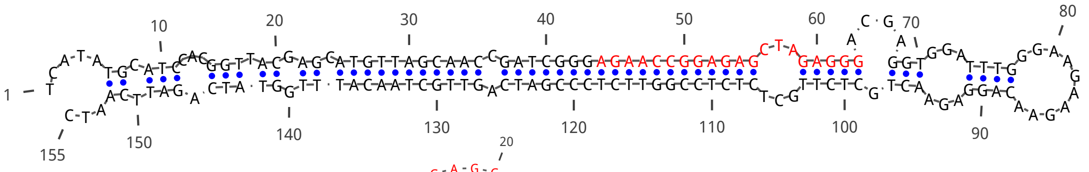

zma-miR06

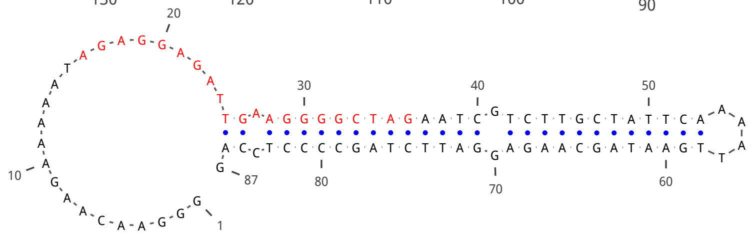

zma-miR07

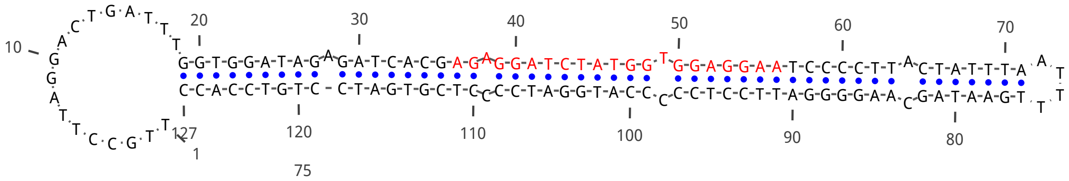

zma-miR08

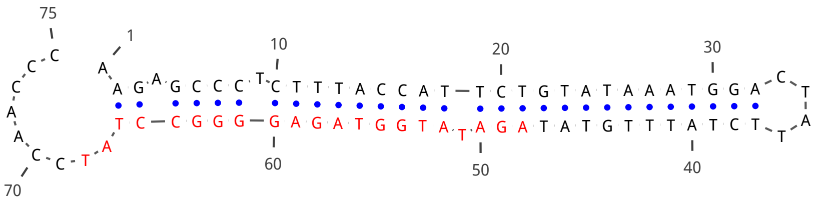

zma-miR09(*)

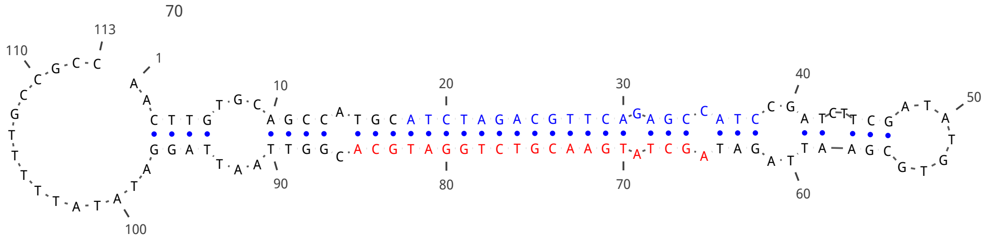

zma-miR10

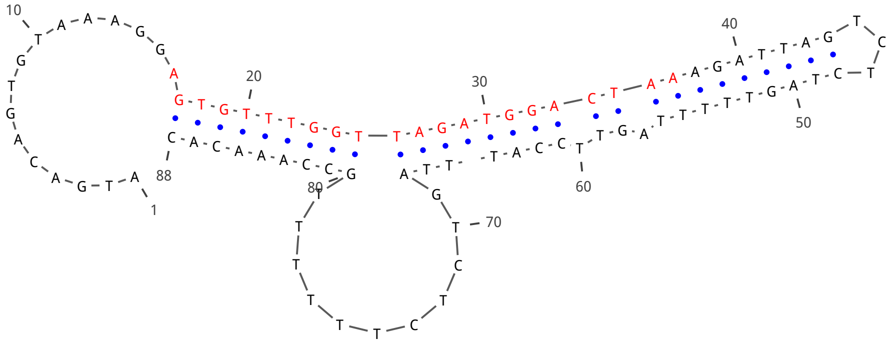

zma-miR11

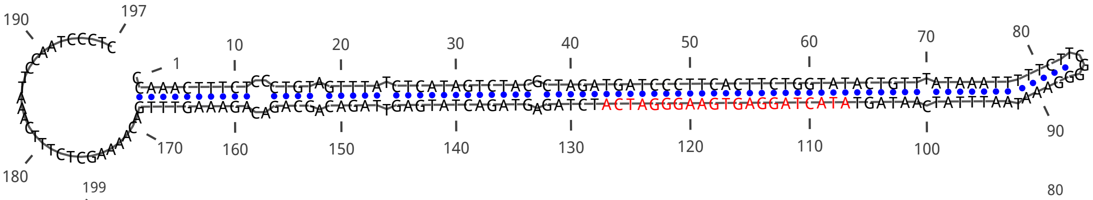

zma-miR12

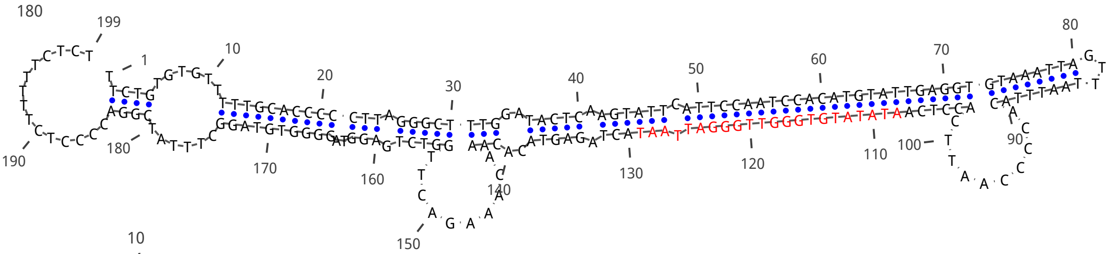

zma-miR13(*)

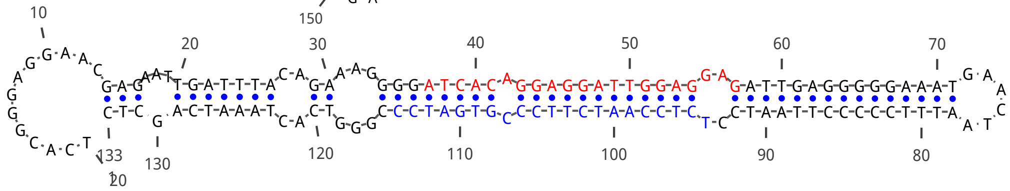

zma-miR14

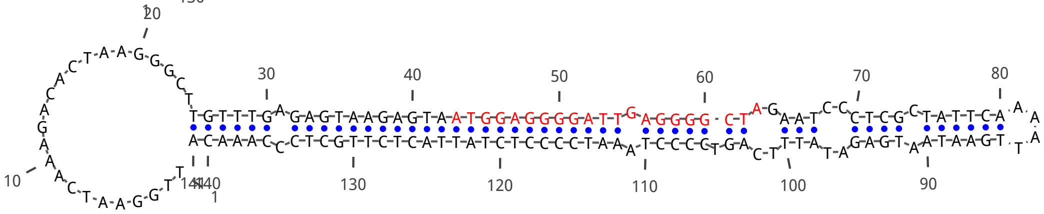

zma-miR15

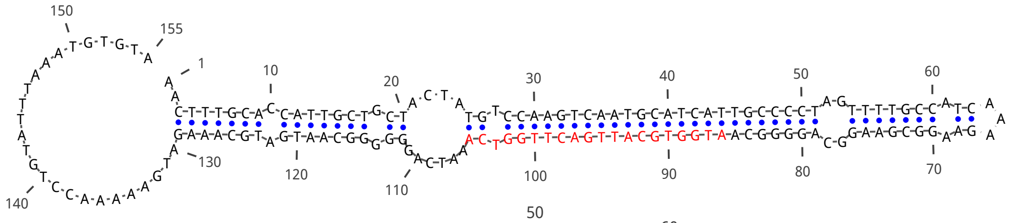

zma-miR16

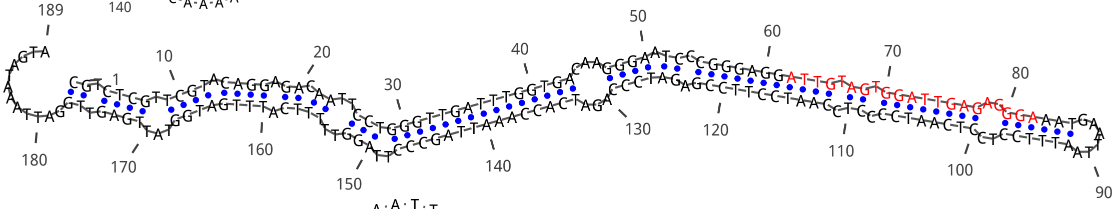

zma-miR17

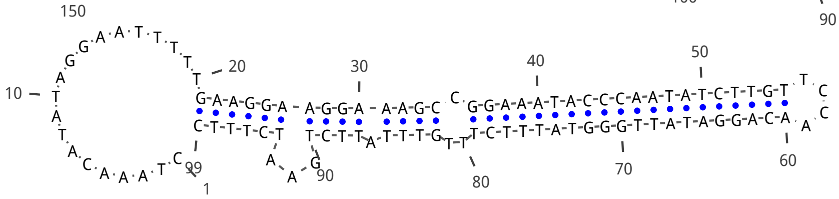

zma-miR18

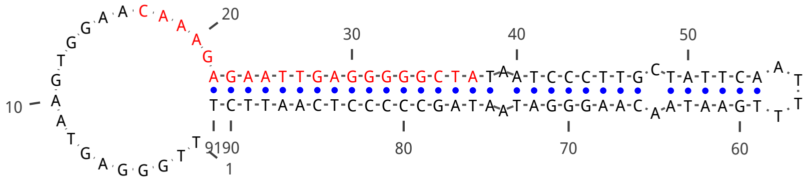

zma-miR19

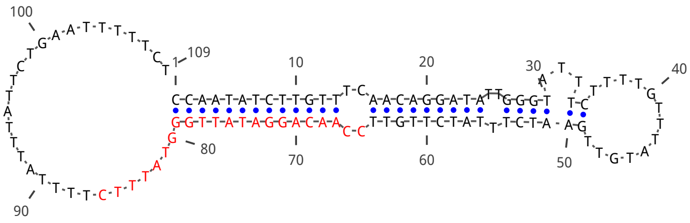

zma-miR20

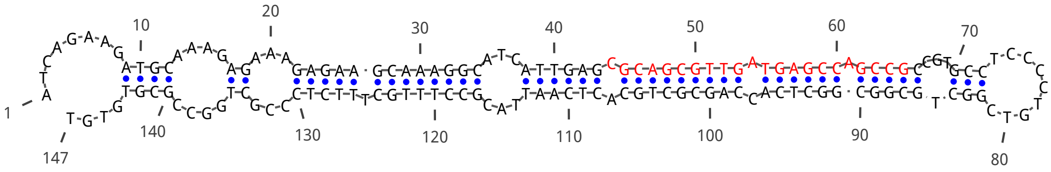

zma-miR21

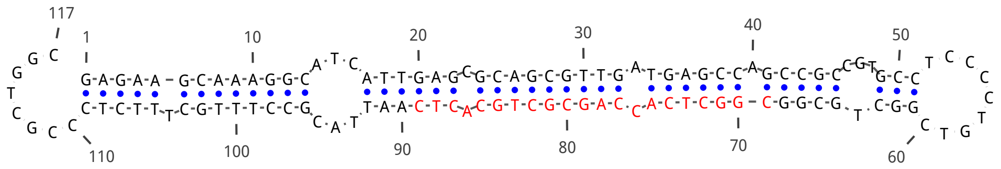

zma-miR22

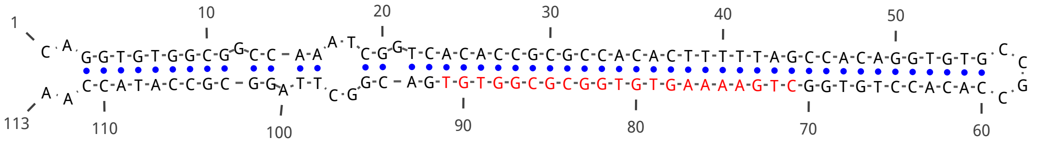

zma-miR23(*)

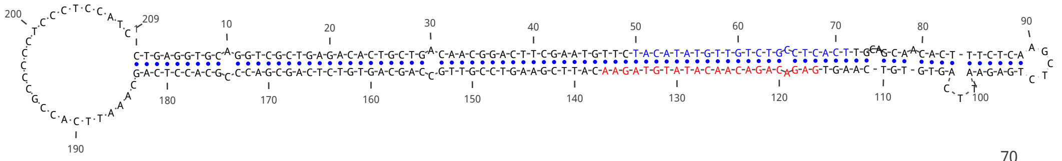

zma-miR24

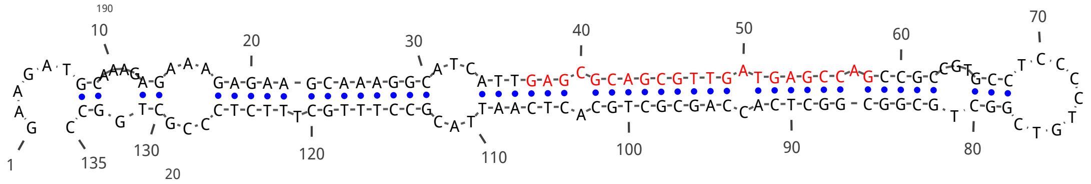

zma-miR25

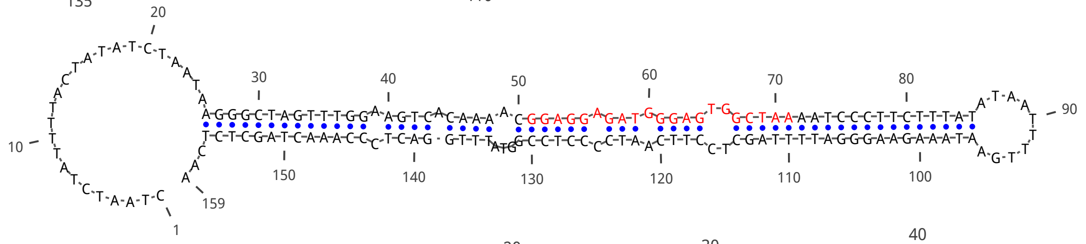

zma-miR26

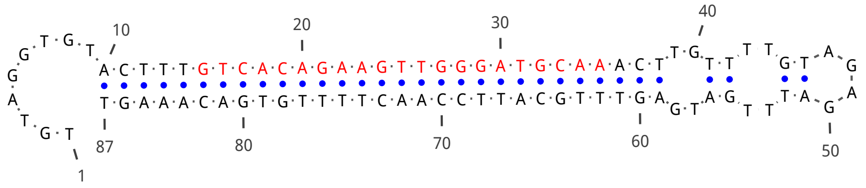

zma-miR27

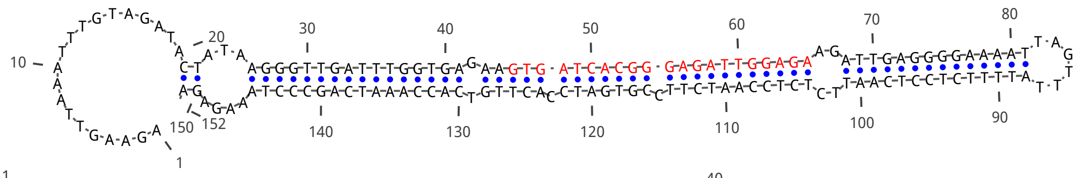

zma-miR28

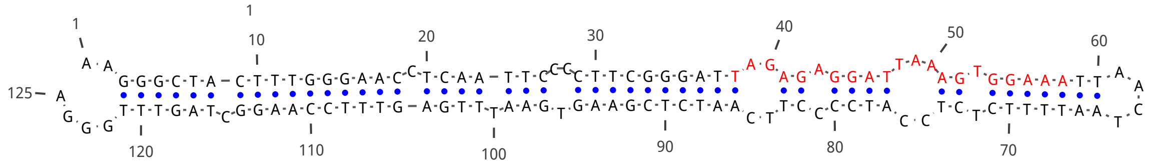

zma-miR29(*)

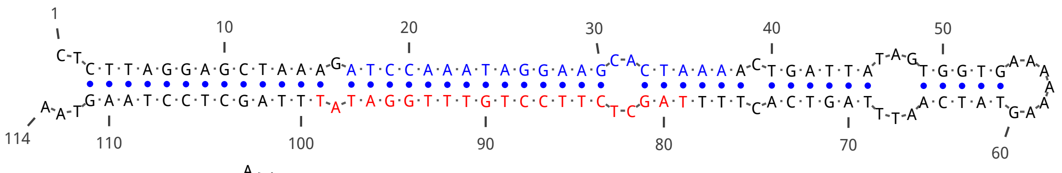

zma-miR30

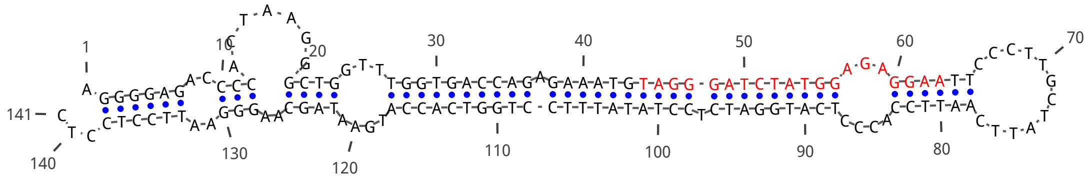

zma-miR31

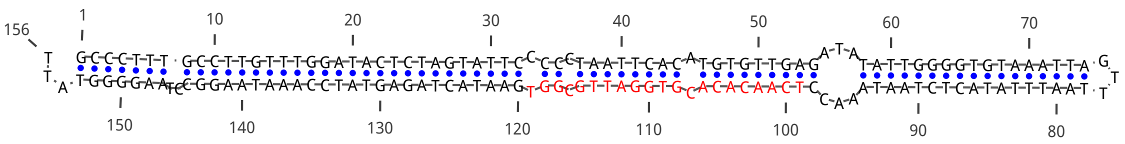

zma-miR32

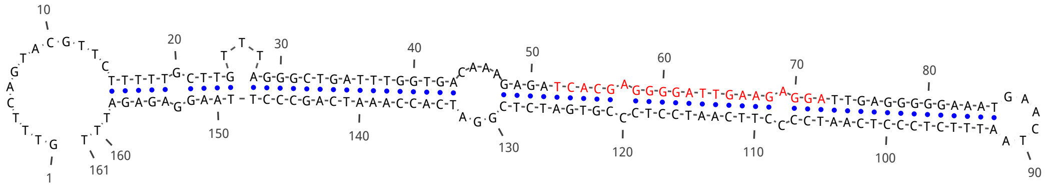

zma-miR33

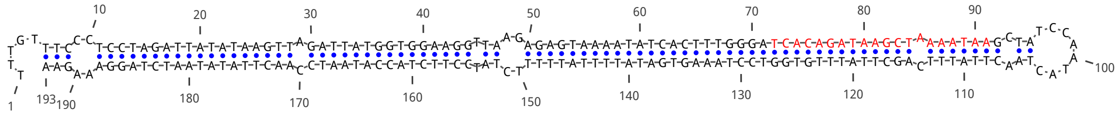

zma-miR34

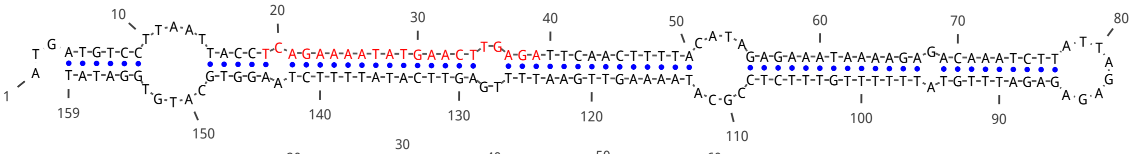

zma-miR35

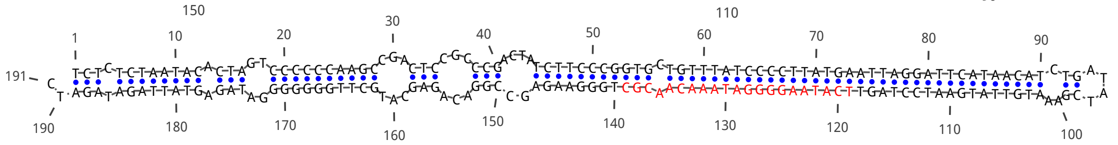

zma-miR36

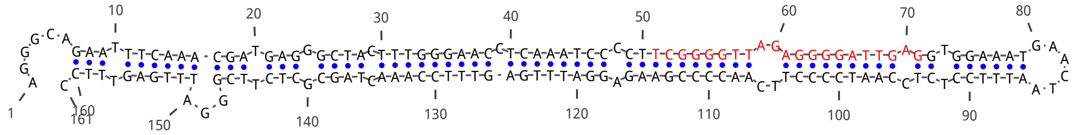

zma-miR37

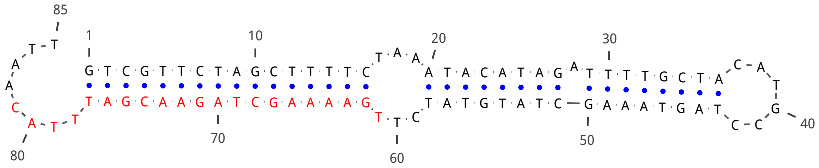

zma-miR38

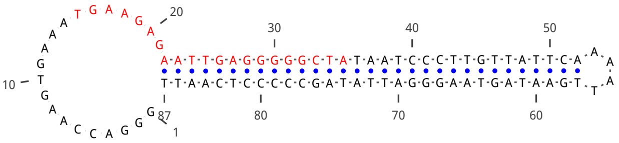

zma-miR39

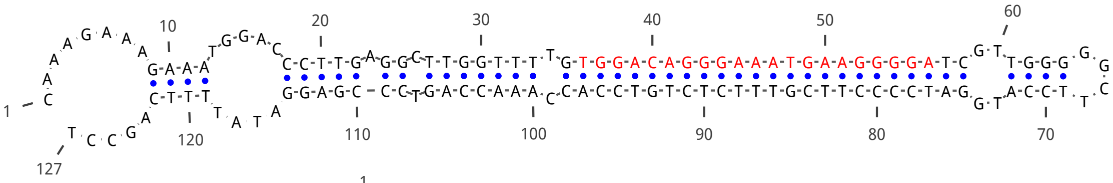

zma-miR40

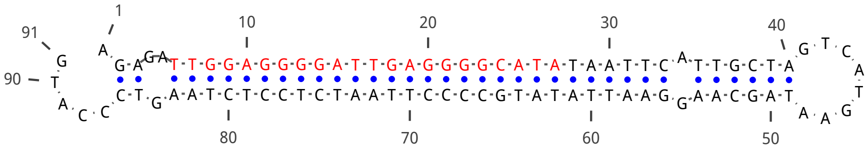

zma-miR41

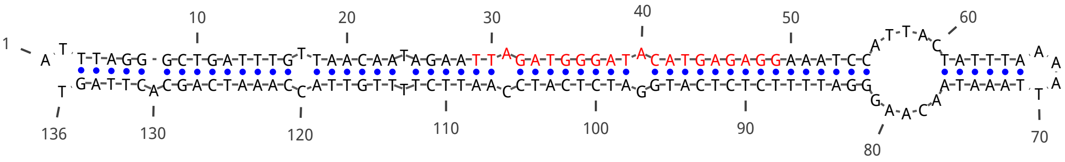

zma-miR42

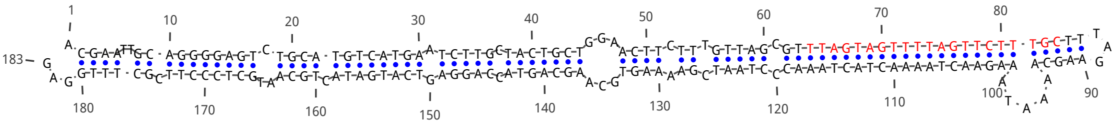

zma-miR43

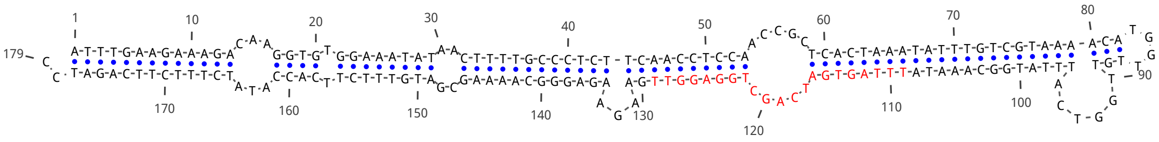

Supplement: Additional file 3 — Secondary structure of novel miRNA precursors. Predicted secondary structures of newly identified miRNAs, mature miRNA sequences were shown in red, while the miRNA* sequences were shown in blue, if any. [file 1471-2164-13-360-S3.pdf]
