# Supplementary material for: Characterization of microRNAs expression during maize seed development
Source: BMC Genomics. 2012 Aug 1;13:360. doi: 10.1186/1471-2164-13-360 (PMC3468377; doi:10.1186/1471-2164-13-360)
Supplement: Additional file 7 — Primers used in this study. [file 1471-2164-13-360-S7.pdf]

Additional file 7

Primers used in this study

```
miR03 RT Primer:      GTCGTATCCAGTGCAGGGTCCGAGGTATTCGCACTGGATACGACTCTCCGGT
miR03 Forward:        GCGGCGGACCGATCGGGAGAA
miR19 RT Primer:      GTCGTATCCAGTGCAGGGTCCGAGGTATTCGCACTGGATACGACGAAATACC
miR19 Forward:        GCGGCGGCCAACAGGATATTG
miR23 RT Primer:      GTCGTATCCAGTGCAGGGTCCGAGGTATTCGCACTGGATACGACTTCTACAT
miR23 Forward:        GCGGCGGGAGACAGACAACAT
miR34 RT Primer:      GTCGTATCCAGTGCAGGGTCCGAGGTATTCGCACTGGATACGACTCTCAAGT
miR34 Forward:        GCGGCGGTCAGAAAATATGAA
Universal Reverse:    GTGCAGGGTCCGAGGT
```
